# Metabolic Changes in Cardiac Aging

**DOI:** 10.31083/j.rcm2403082

**Published:** 2023-03-06

**Authors:** Yan Hao, Wei Liu

**Affiliations:** ^1^Department of Cardiology, The Fourth Affiliated Hospital of Harbin Medical University, 150001 Harbin, Heilongjiang, China; ^2^Department of Geriatric Cardiovascular Division, Guangdong Provincial Geriatrics Institute, Guangdong Provincial People's Hospital, Guangdong Academy of Medical Sciences, 510080 Guangzhou, Guangdong, China

**Keywords:** cardiac aging, metabolism, mitochondria, autophagy, metabolomics, signaling pathways

## Abstract

Cardiac aging is a natural process accompanied by cardiomyocyte hypertrophy and dysfunction. These changes can lead to adverse organ remodeling and ultimately lead to the development of heart failure. The study of cardiac aging is helpful to explore the mechanism of senescence and is of great significance for preventing cardiac aging. Cardiac aging is accompanied by changes in various metabolic functions. In this process, due to the change of metabolic substrates and enzyme activities, oxidative stress response increases, and reactive oxygen species (ROS) increases, accompanied by mitochondrial dysfunction and gene expression changes, so related protein metabolism also changes. Hormone metabolism and autophagy are also involved in the process of cardiac aging. Based on these findings, changes in diet, caloric restriction, improvement of mitochondrial function and promotion of autophagy have been proven to have positive effects in delaying cardiac aging. This article reviews the metabolic changes involved in the process of cardiac aging from different aspects, and briefly reviews the measures to improve cardiac aging.

## 1. Introduction

Cardiovascular disease is the leading cause of morbidity and mortality worldwide, and aging is a significant independent risk factor [1]. As the global population lives longer, age-related cardiac dysfunction and heart failure will become more prominent. Cardiac aging is defined as structural changes and functional deterioration of the heart due to cellular and molecular alterations associated with aging [2]. Cardiac aging is a progressive process characterized by myocardial degeneration, which leads to cell loss, mitochondrial dysfunction, abnormal cardiac remodeling, and ultimately heart failure [3].

**Fig. 1.**
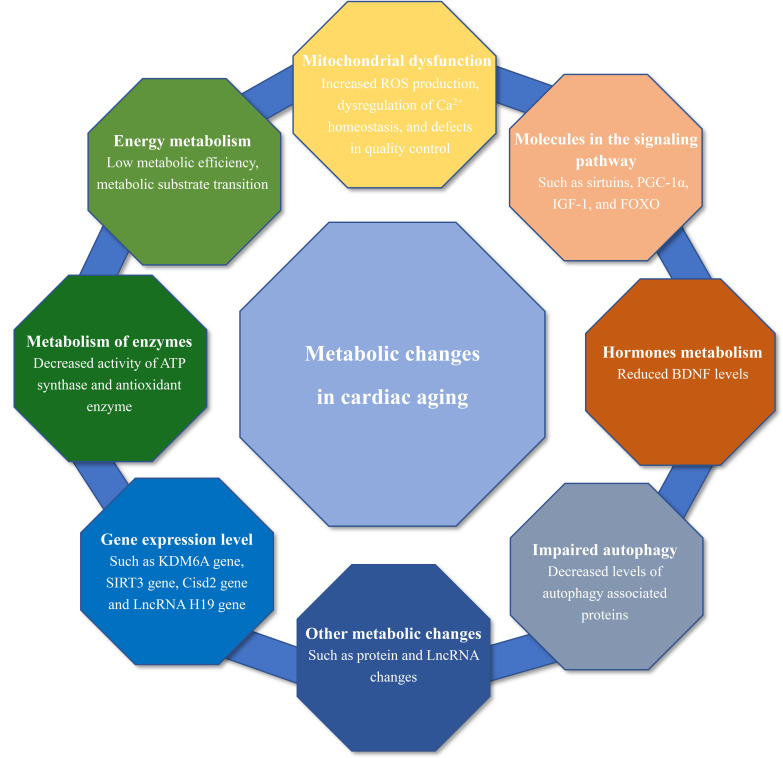
**Overview of metabolic changes in cardiac aging**. ROS, reactive oxygen species; PGC-1α, peroxisome proliferator-activated receptor γ-coactivator 1-α; IGF-1, insulin-like growth factor-1; FOXO, forkhead box O; ATP, adenosine triphosphate; BDNF, brain-derived neurotrophic factor; LncRNA, long non-coding ribonucleic acid.

With the increase of age, the number of cardiomyocytes decreases, the energy transfer efficiency decreases, and the renewal of cardiomyocytes is poor. The function of senescent cardiomyocytes decreases gradually due to the accumulation of more oxidative stress. Compared with normal cardiomyocytes, reactive oxygen species (ROS) levels in senescent cardiomyocytes were significantly increased, and metabolic ability was generally decreased. Cardiac aging involves a variety of metabolic changes (Fig. 1), such as changes in energy metabolism and metabolomics related to mitochondrial dysfunction, and reduced autophagy capacity. In addition, there are age-related changes in hormone secretion levels and aging-induced secretory phenotypes, as well as metabolic changes of signaling molecules related to the regulation of signaling pathways. Some of them are manifested in the process of myocardial senescence, and some in turn accelerate the process of cardiomyocyte senescence and eventually lead to cardiac function disorders. Cardiac aging has gained increasing attention as a potential target for the prevention of cardiovascular diseases, including coronary atherosclerotic heart disease, hypertension, and heart failure [4]. This article reviews the relevant content of metabolic changes in the process of cardiac aging from a new perspective, and summarizes recent research findings. The aim of this work is to better understand the process of myocardial aging and lay the foundation for the prevention of aging.

## 2. Transformation of Energy Metabolism in Senescent Cardiomyocytes

The aging process of myocardium is accompanied by a decrease in the number of ventricular myocytes, which is manifested by a gradual increase in cell volume, down-regulation of organelle function in cardiomyocytes, and accumulation of oxidized proteins and lipids, all of which lead to a gradual decline in normal physiological function of cells. In general, cardiomyocytes show degenerative changes with age, reduced energy transfer efficiency and a gradual decrease in cell number due to apoptosis and necrosis.

The most obvious change in cardiac aging is the shift in energy metabolism substrates. The metabolic activities of cardiomyocytes require a large amount of adenosine triphosphate (ATP). In normal cardiometabolic activities, about 60% of the energy comes from the oxidation of fatty acids, and nearly 40% comes from the oxidation of glucose and lactic acid [5]. However, the amount of ketone bodies and amino acids produced is very small. The supply ratio of various metabolite production capacity of cardiomyocytes is altered by the feeding state and the presence of ischemia or hypoxia. Aging leads to changes in the intracellular environment and gene expression of metabolism-related enzymes, mainly manifested as decreased fatty acid oxidation, and increased glucose utilization due to increased glycolytic related proteins. A change in metabolic substrate preference from fatty acids to glucose and ketone bodies has been observed in hypertrophic or failing cardiomyopathy associated with cardiac aging. Therefore, studies have been conducted to prevent aging by improving dietary status, such as long-term dietary restriction, which significantly reverses age-dependent mitochondrial dysfunction and protects the heart [6]. Caloric restriction (CR) is a dietary pattern that permanently or regularly reduces caloric intake and reliably extends healthy life without causing malnutrition [7]. CR can reduce oxidative stress injury, inflammation and apoptosis, improve telomerase activity and telomere related protein expression, activate autophagy processes and reduce myocardial protein degradation and mitochondrial dysfunction. Therefore, CR has been shown to have a positive effect on improving the function of aging myocardium [8] and caloric restriction mimics are emerging as potential therapeutic agents for cardiovascular diseases. The *CALERIE* study of caloric restriction in humans has demonstrated the positive effects of CR in improving cardiometabolic health and reducing the incidence of cardiovascular disease [9, 10]. The classic ketogenic diet, a high-fat, low-carbohydrate diet, has been found to increase cardiometabolic efficiency and exert protective antioxidant effects on the heart. Exogenous substrates such as ketone bodies, especially β-hydroxybutyric acid (β-HB), can be used to ameliorate adverse metabolic functions of cardiomyocytes due to their potential as alternative energy substrates and their anti-inflammatory and antioxidant properties. At present, β-HB is not only considered as an energy substrate to maintain metabolic homeostasis, but also as a signaling molecule to regulate lipolysis, oxidative stress and neuroprotection. Animal and human studies have confirmed that exogenous ketone supplementation can improve the function of failing heart [11, 12]. Ketogenic diets (KD) have been shown to be involved in the anti-aging process through increased protein acetylation, improved neuroprotection and mitochondrial metabolism, activation of autophagy, and important regulatory roles in signaling molecules and epigenetics [13]. Folic acid is a B-complex vitamin in the form of water-soluble vitamin B9, which inhibits oxidative stress and maintains deoxyribonucleic acid (DNA) stability [14], and attenuates myocardial aging and dysfunction through the endoplasmic reticulum (ER) stress pathway [15]. In addition, some compounds rich in polyphenols or polyamines, such as resveratrol, quercetin, and curcumin, can act as caloric restriction mimics by inducing autophagy and delaying aging [16].

## 3. Altered Metabolic Activity of Enzymes in Senescent Cardiomyocytes

The process of cardiac aging involves changes in the activities of many enzymes, which can interfere with the process of energy metabolism (Table 1). Some evidence supports that oxidative phosphorylation and ATP synthase activity in myocardial mitochondria decrease with increasing age [17, 18]. The aging heart is accompanied by an overall decrease in the activity of antioxidant enzymes, such as manganese superoxide dismutase (MnSOD), which is important in alleviating oxidative stress, and MnSOD levels are significantly lower in aged myocardium compared with young myocardium. The activity of MnSOD in aged myocardium was about 60% of that in young myocardium. In addition, the reduction of antioxidant enzymes and ROS scavenging enzymes will increase the sensitivity to stress responses, which can directly damage DNA and mitochondrial DNA, leading to the high expression of apoptotic factors [19]. This imbalance between oxidation and antioxidants is a common feature of aging in most tissues and organs [20].

**Table 1.** **Metabolic related enzyme and gene changes during cardiac aging**.

		Function	Trends in cardiac aging	Results
Enzyme				
	ATP synthase	Participate in ATP generation	Decreased activity	Interfere with energy metabolism and reduce ATP production
	MnSOD	Alleviate oxidative stress damage	Decreased activity	Increase oxidative stress sensitivity and DNA damage
	AMPK	Regulate biological energy metabolism	Decreased activity	Impair energy metabolism process
Gene				
	*KDM6A*	Regulate demethylation	Down-regulated	Increase cardiomyocyte apoptosis and oxidative stress
	*SIRT3*	Regulate deacetylation	Down-regulated	Reduce the antioxidant stress ability, damage mitochondrial function and autophagy ability
	*Cisd2*	Regulate cytoplasmic ${\mathrm{Ca}}^{2+}$ homeostasis, mitochondrial function, and autophagy	Down-regulated	Damage mitochondrial function, aggravate oxidative stress injury and adverse myocardial remodeling
	LncRNA *H19*	Regulate apoptosis and proliferation of cardiomyocytes	Up-regulated	Accelerate cardiomyocyte senescence

ATP, adenosine triphosphate; MnSOD, manganese superoxide dismutase; DNA, deoxyribonucleic acid; AMPK, adenosine monophosphate-activated protein kinase; LncRNA, long non-coding ribonucleic acid; *Cisd2*, *CDGSH iron sulfur domain 2*.

The activity of citric acid cycle, β-oxidation and oxidative phosphorylation related to mitochondrial productivity will also decrease, thus interfering with the process of energy metabolism. Nicotinamide adenine ${\mathrm{dinucleotide}}^{+}$ (${\mathrm{NAD}}^{+}$), a key coenzyme in mitochondrial oxidative phosphorylation, is the oxidized form of NADH in complex I. At the same time, as the main hydrogen carrier, ${\mathrm{NAD}}^{+}$ plays an important role in the electron transport chain. ${\mathrm{NAD}}^{+}$ stored in mitochondrial matrix can inhibit mitochondrial damage and induce cytochrome C (Cyt C) release [21]. Abnormalities such as altered glucose and lipid metabolism, oxidative stress, and calcium overload can also interfere with cardiac ${\mathrm{NAD}}^{+}$ function. Poly (adenosine diphosphate-ribose) polymerase-1 (PARP-1) is associated with cell death and inflammation during oxidative stress, and loss of sirtuins promotes aging. As an important rebalancing factor in ROS signaling [22], ${\mathrm{NAD}}^{+}$ delays the aging process by regulating the activities of sirtuins and poly (adenosine diphosphate-ribose) polymerases (PARPs) [23].

In addition, the enzyme responsible for cellular energy homeostasis is adenosine monophosphate-activated protein kinase (AMPK), which also regulates mitochondrial ROS production. AMPK activation regulates several biochemical events, including glucose uptake, glycolysis, fatty acid oxidation, and mitochondrial biogenesis. These processes significantly contribute to increasing ATP levels and restoring myocardial contractile efficiency. Aging can impair AMPK signaling pathway, leading to changes in enzyme activity.

A study analyzing left ventricular samples from young and old mice and healthy humans has found that the phosphorylation of carnosine at serine residues S4010 in the elastic N2-B region is altered in mice and elderly human hearts. In the elderly heart, the calcium-activated protease calpain-1 ubiquitinates through the release of carnosine from sarcomeres, showing reduced proteolytic activity and thus impingement of protein quality control, including carnosine, which contributes to reduced myocardial fitness in the elderly [24]. Galectin-3 (Gal-3), a β-galactosidase-binding lectin, is highly increased under various pathological conditions and promotes cardiac remodeling through underlying mechanisms regulating myocardial hypertrophy, inflammation, and fibrosis [25, 26]. It has also been found that lack of Gal-3 during aging exacerbates age-related organ damage. In addition, aging increases the expression of mitochondrial functional proteins and downregulates cardiac autophagy-related proteins and chaperones, which can be reversed by dietary restriction [6].

## 4. Changes in Gene Regulation and Metabolism in Senescent Cardiomyocytes

In the process of aging, cells will suffer from a variety of stresses, leading to changes in gene expression levels, and then to metabolic changes (Table 1). Most of them affect genes encoding proteins involved in oxidative phosphorylation, substrate metabolism and tricarboxylic acid cycle, and transcriptome analysis helps to explain the process and mechanism of aging.

Oxidative stress is one of the main manifestations of aging. Aging changes the functional enrichment of genes related to ROS metabolism. Studies have found that the expression of mitochondria-related genes in aging hearts of humans and rats is differentially altered [27, 28], including genes involved in ROS metabolism in mitochondria. In addition, the rat model showed increased expression of genes associated with oxidase production outside the mitochondria. These changes lead to enhanced ROS production in cardiomyocytes, such as superoxide and lipid peroxidation products, and further increased the sensitivity of aging myocardium to oxidative stress [29]. At the same time, the expression of protein-coding genes related to ROS production and clearance was changed, and the gene encoding the mitochondrial electron transport chain complex I, a site of ROS production was selectively down-regulated in mitochondria. The expression of messenger ribonucleic acid (mRNA) encoding superoxide dismutase (SOD) 1 and SOD2, two major superoxide scavengers in the heart, was also down-regulated in the myocardium of aged rats.

Aging can reduce the expression and activity of lysine demethylase 6A (KDM6A) in human cardiomyocytes. A study on aging mice observed that the expression of *KDM6A* in cardiomyocytes was down-regulated. The loss of *KDM6A* also accelerated the aging of the heart and promoted apoptosis and oxidative stress of cardiomyocytes. This process is achieved by inducing *homeobox C4* (*HOXC4*) to increase ER stress [30]. Oxidative stress, in turn, increases the susceptibility to myocardial injury under stress and promotes interstitial fibrosis and global myocardial dysfunction [31, 32]. Moderate levels of ROS are necessary for myocardial protection, which is achieved by inducing protective signals [33]. However, the imbalance of oxidative stress regulation is an important consequence of ROS production and further affects the life span of organisms. Recent studies have shown that ROS can also accelerate cellular senescence by inducing apoptosis mediated by a variety of intracellular signals [34]. It has been found that serum soluble klotho supplementation can prevent excessive oxidative stress, inflammation, apoptosis and cardiac dysfunction in aging hearts [35].

The expression level of *SIRT3* gene in the myocardium of aged mice was significantly decreased, which increased the level of intracellular acetylation and decreased the ability to resist oxidative stress, and decreased the autophagy of damaged mitochondria, which was not conducive to the renewal of damaged mitochondria [36]. *SIRT3* gene deficiency impaired mitotic phagocytosis, resulting in mitochondrial mitosis and impaired function [37]. One study showed that in ${\mathrm{NAD}}^{+}$ dependent deacetylase *${\mathrm{SIRT3}}^{,-/-}$* mice, loss of *SIRT3* resulted in increased sensitivity of cardiomyocytes to ${\mathrm{Ca}}^{2+}$, resulting in mitochondrial swelling that ultimately affected lifespan and accelerated cardiomyocyte senescence [38]. Similarly, sirtuin-1 (SIRT1) plays a positive role in autophagy and longevity, and can activate AMPK/mammalian target of rapamycin (mTOR) signaling in different pathologies [39].

*Cisd2* is an evolutionarily conserved gene that plays an important role in the regulation of mammalian lifespan and is involved in the regulation of many aging-related pathways, such as sirtuin signaling and autophagy [40, 41]. A decrease in *Cisd2* expression occurs during aging, which leads to mitochondrial dysfunction, disruption of cytosolic ${\mathrm{Ca}}^{2+}$ homeostasis, increased ROS production, and dysregulation of autophagy, manifested as conductance disorders and mechanical contractile dysfunction. One study showed that cardiac *Cisd2* expression was decreased in aged wild type mice, resulting in cardiomyocyte injury, increased interstitial fibrosis, extracellular matrix remodeling, and electromechanical dysfunction. Heart-specific overexpression of *Cisd2* in late life can reverse age-related structural damage and functional disruption and rejuvenate the aging heart [41].

In addition, there are also changes in the expression of other genes during the process of myocardial aging. The expression of long non-coding ribonucleic acid (LncRNA) *H19* was significantly increased in senescent mouse ventricular myocytes and senescent mouse hearts. *H19* acts as an inhibitor of competitive endogenous RNA (ceRNA) by secreting microRNA-19a (miR-19a) to regulate cytokine signaling expression, which subsequently leads to cardiac senescence by stimulating the p53/p21 signaling pathway, while *H19* knockdown inhibits cardiomyocyte senescence [42]. *Gal-3* is closely related to the regulation of cardiac remodeling, and the decreased expression of *Gal-3* gene in the process of aging can aggravate cardiac hypertrophy, fibrosis and apoptosis, increase the expression of Ang II, matrix metalloproteinase-9 (MMP-9) and transforming growth factor β (TGF-β), but decrease the expression of SIRT1 and sirtuin-7 (SIRT7) [43]. Ubiquitin endonuclease activity is also associated with age-related changes in the heart because genes involved in ubiquitin transfer are transcriptional up-regulated with age, which contributes to the induction of cardiomyocyte autophagy.

## 5. Mitochondrial Dysfunction

Myocardial metabolism consumes a lot of ATP, and mitochondria are the main source of cardiac energy metabolism, accounting for about 95% of myocardial ATP [44]. The process of heart aging is accompanied by impaired energy synthesis and decreased function, such as shortened ejection fraction and enlarged left ventricular diameter, while decreased ATP synthesis is also an important cause and predisposing factor of heart failure in aging subjects [19]. There is an abundance of mitochondria in cardiomyocytes and they are more susceptible to energy consumption and oxidative stress. Mitochondrial damage and dysfunction are important factors in many diseases and the aging process itself will lead to changes in mitochondrial structure and number, manifested as mitochondrial swelling, mitochondrial crest sparsity and vacuolar degeneration, decreased activity of respiratory chain complexes, and decreased efficiency of energy transport pathways in mitochondria, resulting in energy metabolism disorders.

The integrity of mitochondrial structure plays an important role in energy metabolism of cardiomyocytes. In cardiomyocytes (CMs), mitochondria form regular “crystal-like” shapes between the myofibrillar lattices, and mitochondrial function in CMs is significantly influenced by the organization of cytoskeletal networks: tubulin, desmin, and cell connexin-folded proteins [45]. In addition, these interactions with cytoskeletal proteins may be directly involved in the regulation of mitochondrial functional behavior by regulating the permeability of the mitochondrial outer membrane (MOM) [46, 47]. In the process of myocardial cell aging, accompanied by metabolic substrate shifts from fatty acids to glucose, this change reduces the production efficiency, leading to peroxide accumulation, thus damage to mitochondrial structure, characterized by highly swollen mitochondria, round, rectangular, or other irregular shapes, with the number of mitochondria cristae decreased significantly. Mitochondria start to lose their strict regular arrangement and uniform distribution, which leads to more focal cavitation occurrence in the mitochondrial matrix. Interference in the connection between mitochondria and cytoskeletal filaments and structural integrity directly affects the subcellular localization of mitochondria and the efficiency of mitochondria-ATPase feedback signaling [48]. Trimetazidine can increase the efficiency of glucose oxidative metabolism, thereby reducing the damage to mitochondrial structure caused by peroxide and improving myocardial senescence. In addition, oleanolic acid (OA) treatment was found to rescue mitochondrial ultrastructural abnormalities (loss of myofilament alignment, mitochondrial swelling, and increased roundness) and mitochondrial biogenesis caused by aging [49].

Age-related mitochondrial dysfunction is also evident at the functional level of the aged heart, including increased ROS production, dysregulation of ${\mathrm{Ca}}^{2+}$ homeostasis, and defects in quality control.

Mitochondria are the main organs producing ROS, so they are also the most vulnerable to oxidative damage, which leads to the continuous production of ROS and the vicious cycle of mitochondrial dysfunction. Excessive oxidative stress results in mitochondrial organelle damage and protein aggregation [50]. The ability of mitochondria to produce ${\mathrm{NAD}}^{+}$ in senescent cardiomyocytes is reduced, accompanied by mitochondrial DNA damage. Defects in autophagy and lysosomal dependent degradation pathways also play a key role in the development of dysfunctional organelles [41]. In addition, oxidative stress alters the cascade in signaling pathways involved in aging and autophagy. Thus, oxidative stress causes the heart to age [51].

ROS produced by oxidative phosphorylation of mitochondria during cardiac aging can act on different protein targets, including electron transport chains and bridging proteins in the inter-tissue space, and ultimately disrupt the close association between mitochondria and sarcoplasmic reticulum (SR), resulting in reduced calcium uptake. Mitochondrial regeneration of electron donor NADH and antioxidant nicotinamide adenine dinucleotide phosphate (NADPH) in cardiomyocytes is inhibited under conditions of increased heart rate (such as exercise, β-adrenergic stimulation) due to decreased calcium exchange, which manifests as an uncoupled bioenergetic feedback response, resulting in a consequent increase in mitochondrial ROS production [52]. Age-related reductions in ${\mathrm{Ca}}^{2+}$ retention in interfibrous mitochondria could explain the increased susceptibility of aged myocardium to stress-induced cell death. Aging also leads to the disturbance of the electron transport chain in the mitochondria between fibers in myocardial tissue, and the mitochondrial membrane potential of aged cardiomyocytes decreases more rapidly, thus causing dysfunction.

Mitochondrial dysfunction can also lead to myocardial aging. Cardiac aging is accompanied by cardiac hypertrophy and fibrosis, which increases the susceptibility of cardiomyocytes to stress. Therefore, mitochondrial dysfunction caused by oxidative stress is considered to be an important cause of cardiac aging and heart failure. One of the most characteristic mechanisms of aging caused by mitochondrial dysfunction is the excessive by-products of ROS during respiration. Additionally, other mitochondrial mechanisms such as mitochondrial calcium homeostasis, mitochondrial quality control mechanisms, and mitochondrial dynamics are also involved in the establishment of premature senescence [53]. Some studies have shown that the concomitant reduction of ${\mathrm{NAD}}^{+}$ during myocardial aging is closely related to the opening of mitochondrial permeability transition pore (mPTP) in response to stress. Decreased mitochondrial ${\mathrm{NAD}}^{+}$ stocks may be an important inducer of cardiomyocyte senescence, manifested by reduced adaptation to adverse homeostasis changes, such as high glucose, hypoxia, drug stress, or ischemia-reperfusion injury [54]. By reducing the efficiency of mitochondrial oxygen consumption, the mitochondrial repair mechanism is further decreased, thus forming a vicious cycle [55]. The decrease of myocardial function is also caused by the decrease of energy transfer efficiency, especially the creatine kinase (CK) pathway [48].

Mitochondria are closely related to myocardial senescence and play an important role. Therefore, targeted therapy against mitochondria is of great significance for improving aging-induced cardiomyopathy. Polyamines are involved in a wide range of cellular processes, including autophagy mitochondrial quality control, anti-inflammatory responses, and protection against oxidative stress. Some studies have found that injection of spermine (Spm) and spermidine (Spd) can prevent cardiac dysfunction, improve mitochondrial function, and down-regulate cell apoptosis [56]. Furthermore, increasing the expression of mitochondrial metabolic enzymes can enhance fatty acid oxidation and reduce glucose energy supply, thereby improving mitochondrial biogenesis function [57].

## 6. Metabolomic Changes in Signaling Pathways

The senescence process of cardiomyocytes is accompanied by the decrease of some metabolic related regulatory factors. Therefore, metabolomic study of the signaling pathways related to cardiac aging may provide new therapeutic targets for delaying the occurrence of aging (Table 2).

**Table 2.** **Metabolomic changes in signaling pathways during cardiac aging**.

Signaling pathways	Metabolic changes	Results
SIRT1/PGC-1α	SIRT1 expression in heart tissue decreases in an age-dependent manner, resulting in decreased activation of downstream PGC-1α.	It exacerbates aging by accelerating ROS accumulation and triggering oxidative damage to lipids, proteins, and DNA.
PI3K/AKT/FOXO	PI3K/AKT signaling is activated in a cascade, leading to phosphorylation of FOXO and inhibition of its transcriptional activity.	Mitochondrial fatty acid oxidation pathways and FOXO-mediated transcription of nuclear-encoded mitochondrial genes are inhibited.

SIRT1, sirtuin-1; PGC-1α, peroxisome proliferator-activated receptor γ-coactivator 1-α; ROS, reactive oxygen species; DNA, deoxyribonucleic acid; PI3K, phosphoinositide 3-kinase; AKT, protein kinase B; FOXO, forkhead box O.

Aging itself as a kind of stress that can activate the activity of sympathetic nerves and aggravate the occurrence of oxidative stress response. Excessive oxidative stress leads to damage of cellular components (including DNA, proteins, and lipids), myocardial remodeling, and heart failure [58]. As mentioned above, sirtuins are a family of enzymes composed of ${\mathrm{NAD}}^{+}$ dependent histone/protein deacetylases, which can regulate cell stress, metabolism, aging, and apoptosis, and play a role in anti-stress and delaying cell senescence. During cardiac aging, the expression and activity of sirtuins gradually decrease, leading to a decrease in the heart’s resistance to disease. Activation of SIRT1 enhances mitochondrial biogenesis through its downstream protein peroxisome proliferator-activated receptor γ-coactivator 1-α (PGC-1α), thereby supplementing metabolic signaling pathways and suppressing inflammatory signaling [59]. PGC-1α is a strong transcriptional coactivator of transcription factors and nuclear receptors as well as major regulator of mitochondrial biogenesis and oxidative phosphorylation [60]. Compared with young myocardium, the expression, deacetylation and activity of PGC-1α are lower in aged myocardium [61]. SIRT1 can promote mitochondrial biogenesis and function through PGC-1α deacetylation, and SIRT1 expression in heart tissue also decreased in an age-dependent manner. Disruption of mitochondrial biogenesis slows organelle turnover and exacerbates aging by accelerating ROS accumulation and triggering oxidative damage to lipids, proteins, and DNA [62]. Spermidine was found in a study to improve cardiomyocyte aging by activating SIRT1/PGC-1α signaling pathway, thereby enhancing mitochondrial biogenesis and function, which provided a new therapeutic strategy to combat cardiac aging and prevent age-related cardiovascular diseases [63].

Insulin/insulin-like growth factor (IGF) signaling pathway is closely related to aging [64]. IGF-1 can induce DNA damage and increased ROS production, and enhance cell senescence through the p53 pathway. In a long-term follow-up study of the elderly population in the community, insulin-like growth factor-binding protein-7 (IGFBP7) levels were found to correlate with structural changes in the aging heart muscle and independently predicted cardiovascular disease risk [65]. In the presence of insulin and/or IGF-1 signaling, phosphoinositide 3-kinase (PI3K)/protein kinase B (AKT) signaling is activated in a cascade, leading to phosphorylation of forkhead box O (FOXO) and inhibition of its transcriptional activity [66]. One study showed that myocardial aging from compensatory hypertrophy to heart failure is accompanied by increased AKT signaling and decreased FOXO1 levels [67]. FOXO activates damage repair mechanisms and plays a key role in regulating substrate utilization and oxidation in the heart. Additionally, FOXO also acts downstream of AKT. FOXO has also been shown to regulate phosphorylation of AKT itself, thereby controlling insulin sensitivity and glucose uptake in the heart. Sustained activation of AKT in the heart can inhibit the mitochondrial fatty acid oxidation pathway or act synergistically with other transcriptional regulators by reducing FOXO-mediated transcription of nuclear-encoded mitochondrial genes. Resveratrol is a SIRT1 activator that improves cardiomyocyte function by promoting FOXO1 transcription and reversing this process [68].

mTOR is a serine/threonine kinase in the PI3K family. mTOR interacts with other subunits to form two different complexes (mTORC1 and mTORC2), which are involved in the regulation of aging by regulating metabolic adaptation, autophagy and mitochondrial biogenesis. mTORC1 plays a role in regulating cardiac development and structural stability. Growth factors stimulate mTORC1 activity by activating the lipid kinase PI3K, which regulates cell growth and cell size by regulating translation, nucleotide biosynthesis, lipogenesis, glycolysis, and autophagy [69]. mTOR signaling is abnormally activated during aging, and rapamycin can increase autophagy by inhibiting mTOR-mediated phosphorylation of UNC51-like kinase 1 (Ulk-1) (a key regulator of autophagosome formation) [70], thus conducive to the extension of life [71].

## 7. Changes in Metabolism of Hormones During Cardiac Aging

There is evidence that pre-atrial natriuretic peptide (ANP) levels are reduced in the atria of older rats and that aging impairs ANP production, leading to heart failure and hypertension. In addition, ANP variants affect cardiovascular responses to exercise in older adults. Bradykinin can promote the activation of endothelial nitric oxide (NO) synthase and protect endothelial cells from cellular senescence, and up-regulate the activity and expression of antioxidants Cu/Zn-SOD and MnSOD, and down-regulate the activity of NADPH oxidase, then inhibit the production of ROS, and finally protect cardiomyocytes from oxidative stress-induced senescence [72].

One study found that in older rats, cardiac expression of glucocorticoid receptor (MR) was higher than adolescent rats and accompanied by increased expression of p53 and decreased expression of PGC-1α, however, elderly rats had mitochondrial changes which increased oxidative stress and reduced SOD. *In vitro* experiments also confirmed that MR selective antagonism can partially delay myocardial aging. This study showed that the up-regulation of glucocorticoid receptors during aging is associated with mitochondrial damage and leads to cardiac dysfunction.

Brain-derived neurotrophic factor (BDNF) is a pleiotropic protein secreted/expressed in multiple body sites, including blood vessels and smooth muscle cells, skeletal muscle, platelets, and especially the heart, which can be secreted by cardiomyocytes [73]. BDNF governs autonomic transmission to the heart and exerts prominent angiogenic effects [74]. BDNF directly regulates myocardial mechanical function under normal and disease conditions through stimulation of cardiac tropomyosin-like receptor kinase B (TrkB), so BDNF/TrkB stimulation is essential to optimize basal cardiac contraction and relaxation. Furthermore, BDNF acts directly on ${\mathrm{Ca}}^{2+}$ cycling in a calmodulin-dependent protein kinase II-dependent manner and can be altered during old age and consequently lead to cardiac autonomic fiber poverty [75]. The physiological aging process is accompanied by a decrease in BDNF and eventually leads to structural and functional impairment of autonomic nervous system (ANS). As a stress, aging itself can lead to the disorder of sympathetic nervous system (SNS), such as increased circulating catecholamine level and the dysfunction of cardiac β-adrenergic receptor (β-AR) signaling, decrease the circulating level of BDNF, damage autonomic nerve fibers, and increase the incidence of cardiovascular diseases. On the other hand, autonomic fiber remodeling desensitizes/dysfunctions and downregulates cardiac β-AR expression, accompanied by a decrease in parasympathetic response [76, 77]. The loss of active BDNF results in significant impairment of cardiac function during aging and predisposes the elderly to cardiovascular diseases of different nature and etiology. Decreased circulating levels of BDNF leads to impaired heart function and the progression of heart disease [78]. Because decreased cardiac β-AR reactivity leads to decreased cardiac function and muscle strength reserve, treatments to restore β-AR reactivity (such as beta blockers) can improve cardiac function in older patients [79]. To reduce sympathetic nerve activity, studies have found that metoprolol can down-regulate arginine vasopressin (AVP) induced acetylated p53 and p21 expression, so metoprolol can protect AVP induced cardiomyocyte senescence [80].

## 8. Impaired Autophagy

Mitochondria are in dynamic change, and their homeostasis is regulated by mitotic phagocytosis, including biogenesis and mitophagy [81]. Mitotic phagocytosis is a selective degradation process of damaged or stressed mitochondria and thus has an important protective effect on aging myocardium. The aging process is accompanied by the enlargement of mitochondria, which may be caused by the reduction of mitochondrial dynamin related protein-1 (DRP-1) mediated division [82], thus reducing mitophagy function. The inhibition of autophagy activity is due, at least in part, to reduced levels of key autophagy-related proteins. As mitochondrial proteins synthesized by nuclear genes are continuously introduced into existing mitochondria, this will lead to damage to mitochondria by ROS and reduce ATP production.

In aged heart, there is an imbalance between labeling and degradation steps in the aged myocardium due to reduced autophagosome formation. It involves multiple molecular pathways, such as mTORC1, AMPK, sirtuins, FOXO, and ROS. Mitochondria, ER, peroxisomes and proteins damaged by oxidative stress are degraded and recycled through autophagy to slow cell death. A moderate amount of ROS can induce autophagy, but excessive ROS can inhibit autophagy and aggravate protein aggregation and mitochondrial function damage, leading to increased ROS generation, forming a vicious cycle. Interventions that regulate autophagy and oxidative stress can reverse cardiac aging [83].

Recent studies have shown that the expressions of *Atg5*, *Atg7*, and *Beclin1* genes associated with autophagy in aged myocardium are decreased. Decreased cardiomyocyte autophagy in aging hearts is associated with dysregulation of PI3K/Akt/mTOR, AMPK and/or SIRT1 signaling pathways. Also, ROS and neurohormones such as endothelin-1 (ET-1) mediate the reduction of cardiomyocyte autophagy during cardiac aging. The regulation of cardiomyocyte autophagy may provide new strategies for the prevention and treatment of senile cardiomyopathy. As previously described, rapamycin enhances autophagy and promotes cardiomyocyte survival by inhibiting the Akt/mTORC1 pathway [84]. Moreover, studies have shown that metformin can activate cardiac autophagy and improve cardiac function in diabetic mice through an AMPK-dependent mechanism [85].

## 9. Molecular Mechanisms and Signaling Pathways of Age-Related Myocardial Remodeling

**Fig. 2.**
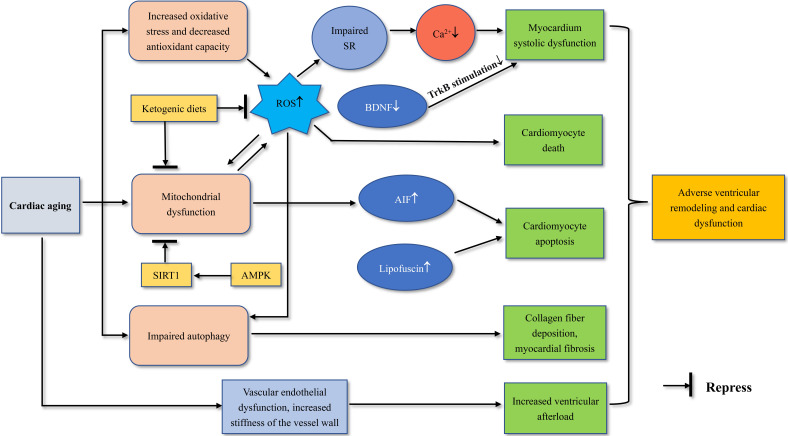
**Mechanisms of age-related myocardial remodeling**. SIRT1, sirtuin-1; AMPK, adenosine monophosphate-activated protein kinase; ROS, reactive oxygen species; SR, sarcoplasmic reticulum; BDNF, brain-derived neurotrophic factor; AIF, apoptosis-inducing factor; TrkB, tropomyosin-like receptor kinase B.

With the increase of age, the senescent heart shows myocardial hypertrophy, interstitial fibrosis and impaired systolic function (Fig. 2). In healthy people, the heart gradually develops diastolic dysfunction with age, increasing the incidence of heart failure [86]. Similarly, decreased longitudinal systolic function is also associated with cardiac aging, which may be related to the occurrence of heart failure with preserved ejection fraction [87]. Oxidative stress and changes in energy metabolism trigger hypertrophic and pro-fibrotic signaling cascades, resulting in cell death and progressive cardiomyocyte loss. Lipofuscin has a progressive inhibitory effect on autophagy during aging. The crosslinked polymer lipofuscin was not degraded by lysosomal hydrolases, and the accumulation of lipofuscin induced cardiomyocyte apoptosis. Apoptosis-inducing factor (AIF) is a factor that induces cysteine proteinase-dependent apoptosis, and cardiac mitochondrial dysfunction may lead to increased AIF level in myocardial nucleus, which may also lead to cardiomyocyte apoptosis [88]. To compensate for cell loss, the remaining cardiomyocytes undergo hypertrophy. In addition, the aging process is accompanied by increased intimal thickness and collagen deposition, which thickens and stiffens the arterial wall, leading to the development of left ventricular hypertrophy due to increased afterload and vessel wall stress.

AMPK is a major regulatory kinase directly involved in many metabolic processes, including fatty acid oxidation and glycolysis, and can modulate the SIRT, mTOR, and PGC-1α signaling pathways. Lack of AMPK promotes aging-related cardiac hypertrophy [89]. AMPK is a major target of metformin, which can activate AMPK to protect the heart from aging-induced cardiac hypertrophy [90], and improve cardiac function in aging. Recent studies have shown that the anti-hypertrophy effect of metformin is also associated with the prevention of mitochondrial dysfunction by the SIRT1/endothelial nitric oxide synthase (eNOS)/p53 pathway [91]. The aging process is accompanied by the decrease of sirtuin-2 (SIRT2) expression. Studies have shown that SIRT2 can inhibit aging-related myocardial hypertrophy through the signaling of liver kinase B1 (LKB1)-AMPK pathway [92]. In addition, FOXO protein reduces insulin sensitivity and inhibits cardiac hypertrophy by inhibiting calcineurin. SIRT2 can affect microtubule stability through tubulin deacetylation and improve cardiac hypertrophy by regulating FOXO signaling [93]. Sirtuin-3 (SIRT3), as a mitochondrial sirtuin isoform, can stimulate oxidative phosphorylation by direct deacetylation of electron transport chain complexes. Overexpression of SIRT3 promotes autophagy and reduces cardiac hypertrophy. Cyclophilin is a protein that regulates mitochondrial permeability transition pore opening and prevents the adverse effects of cardiac aging. SIRT3 also inhibits pathological cardiac hypertrophy by deacetylating cyclophilin [94]. Several natural and synthetic small molecule inhibitors of acetyltransferase p300, including curcumin and the p300 activity regulator resveratrol, have been used to prevent or treat adverse remodeling in aging myocardium [95].

Myocardial fibrosis during aging is associated with the accumulation of collagen in the extracellular matrix. Aging increases the rate of ventricular collagen turnover and deposition in fibroblasts, which is manifested by increased collagen content, decreased collagen solubility and increased collagen cross-linking. Oxidative damage to the calcium pump in the SR due to increased oxidative stress in aging cardiomyocytes leads to impaired ${\mathrm{Ca}}^{2+}$ cycling/processing, and changes in the active diastolic properties of myocytes, leading to delayed ventricular diastole [96].

An experiment in mice showed that a continuous KD also improved poor left ventricular remodeling and the development of myocardial dysfunction [97]. This may be related to the decrease of mitochondrial ROS production, the increase of mitochondrial ATP and membrane potential, and the promotion of autophagy [97]. Similarly, rapamycin also plays a positive role in promoting autophagy to prevent aging-induced ventricular remodeling [98].

## 10. Other Metabolic Changes

During the developmental stage, embryonic CMs rely on glycolysis to produce ATP. As the heart grows, CMs undergo metabolic changes from anaerobic glycolysis to oxygen-dependent mitochondrial oxidative phosphorylation, and the production of ROS leads to DNA damage and cardiac cell cycle arrest. As the heart ages, myocardial degeneration occurs, leading to cardiomyocyte death, but there is currently evidence to support regeneration of the aged myocardium, with apoptotic cardiac cells being replaced by new cells derived from cardiac stem/progenitor cells (CSCs/CPCs) [99]. The adult mammalian heart contains a large amount of endogenous CSCs, which is clonogenic, self-renewing and pluripotent. CSCs were involved in the response to cardiac injury and physiological CMs transition during the life cycle, and have a significant capacity for cardiac tissue regeneration [100]. Reactivation of developmental signaling factors in the heart leads to metabolic reprogramming of CMs, which favors increased cell-cycle activity and myocardial repair after injury. Glycolysis is the preferred energy generation pathway for proliferative CMs [101]. Overexpression of pyruvate kinase muscle isoenzyme 2 (Pkm2) is associated with increased glycolytic flux and enhanced biosynthetic pentose phosphate pathway, which is essential for cell growth and proliferation. It has been shown that inhibition of fatty acid utilization can promote cardiomyocyte proliferation in the heart, and reintroduction of Pkm2 in adult hearts can enhance CMs proliferation, cardiac function, and long-term survival [102].

Cell senescence is accompanied by changes in protein levels, and some proteins are involved in the mechanism of cardiomyocyte senescence and thus promote the occurrence of senescence. In one study, proprotein convertase subtilisin/kexin type 6 (PCSK6) protein expression was significantly decreased in D-galactose-induced senescent rat embryonic cardiomyocytes. Using *PCSK6* knockout animal model, it was confirmed that the loss of PCSK6 protein increased the expression levels of P16 and P21, as well as the β-galactosidase activity associated with aging, which was manifested as the increase of ROS and apoptosis, resulting in functional injury of cardiomyocytes [103]. Overexpression of PCSK6 prevents this phenotype, improves cell function, and inhibits ER stress in cardiomyocytes. The above studies indicate that PCSK6 can mediate ER stress response and regulate cardiomyocyte senescence [103]. It has been found that the level of angiotensin-converting enzyme 2 (ACE2) protein in the heart tissue of aged mice is lower than that of young mice, and the knockout of *cystathionine gamma lyase* (*CSE*) gene can induce moderate oxidative stress in the heart of mice and further inhibit ACE2 protein level. Incubation of rat cardiomyocytes with low dose of hydrogen peroxide ($H_{2}$$O_{2}$) could inhibit ACE2 protein levels and induce cell senescence, while co-incubation with NaHS (H2S donor) could completely reverse ACE2 protein senescence [104].

A study that analyzed the cardiac glycoproteome of mice of different ages by western blot and matrix-assisted laser desorption ionization time-of-flight (MALDI-TOF) found that high mannose N-glycans increased with age, and guanosine diphosphate (GDP)-mannose pyrophosphorylase B (GMPPB) could promote the supply of GDP-mannose. This study showed that there are changes in glycosylation mechanisms during myocardial aging, which are also concomitant protein changes in the pathways associated with aging [105]. A study using a mouse model of natural aging found increased proton leakage in mitochondria of aging hearts, revealing excess proton leakage as a novel mechanism of age-related cardiac dysfunction that could be reversed using SS-31 [106].

Circular RNAs (circRNAs) are involved in glucose metabolism, fatty acid oxidation, mitochondrial biosynthesis and other biological processes, and they are also associated with myocardial ischemia and cardiac aging related diseases [107]. MicroRNA (miRNA) are related to gene expression regulation, involved in the gene regulation of left ventricular structure and function during human aging, and can be used as biomarkers for age-related cardiac risk prediction [108]. In induced senescent cardiomyocytes, senescence-mitophagy associated LncRNA (LncR-SMAL) was increased in both cytoplasm and nucleus of cardiomyocytes, which indicated that LncR-SMAL was an up-regulated LncRNA in elderly hearts. Overexpression of LncR-SMAL resulted in decreased diastolic function and significantly increased protein levels of aging marker genes *p53* and *p21*. Most senescent cells were accompanied by significant activation of senescence-associated secretory phenotype (SASP). SASP activation is a dynamic, cell type-dependent process that can influence the surrounding cellular microenvironment and drive body disorders. Cardiac aging is associated with up-regulation of the SASP. In some studies, compared with healthy hearts, LncR-SMAL overexpressing hearts showed a significant increase in SASP [109]. LncR-SMAL and mitophagy function have therapeutic potential in the treatment of cardiac aging. The decrease of LncR-SMAL can prevent cardiomyocyte senescence, which is mainly achieved by promoting mitotic phagocytosis of cardiomyocytes and maintaining mitochondrial quality control.

## 11. Prevention and Treatment

The study of metabolic changes in the process of cardiac aging is helpful to explore the prevention and treatment of myocardial diseases in the elderly. As previously mentioned, CR, as a repeatable dietary intervention, plays a positive role in improving myocardial metabolism, alleviating oxidative stress damage [110] and inducing autophagy [111]. Clinical evidence has shown that CR is an effective treatment for inhibiting cardiac aging and improving cardiac remodeling. Phenolic compounds (PC) have a protective effect on the heart, and a study has shown that long-term consumption of PC can improve the function of aging hearts through antioxidant effects and reduce the occurrence of adverse ventricular remodeling [112]. In addition, resveratrol, as a SIRT1 activator, has also been shown to have a cardioprotective effect in regulating aging-related oxidative homeostasis and reducing inflammatory responses [113]. β-AR desensitization during cardiac aging leads to sympathetic dysregulation. High-intensity training increased the density of β-AR in the hearts of older rats, thereby enhancing the responsiveness to adrenergic stimulation. Exercise also increases antioxidant capacity by increasing reactive oxygen scavenging enzymes. Therefore, exercise as an induced form of physiological stress can potentially slow down or reverse the process of cardiac aging [114].

At present, there are also some drugs in clinical application, showing positive effects on the treatment of cardiac aging. Rapamycin can activate AMPK pathway, inhibit mTOR pathway, induce autophagy and promote mitochondrial biogenesis [115], and is widely regarded as the compound with the greatest influence on longevity [116]. At the same time, the use of rapamycin can also improve the diastolic dysfunction in the aging process of the heart [57]. Spermidine treatment attenuates the aging process by activating autophagy, and epidemiological analyses of a large human cohort have also shown that increased dietary spermidine intake is associated with reduced cardiovascular death and longer lifespan [117]. As mentioned in the previous section, CPCs show potential therapeutic value in repairing damaged senescent cardiomyocytes. Pim-1 is a conserved serine/threonine protein kinase that protects myocardium through anti-apoptotic effects [118], and its expression is reduced in CPCs. Overexpression of pim-1 by gene modification can delay senescence and improve the function of injured myocardium [119]. Although studies have shown that transplantation of CPCs in senescent rats shows improvement in cardiac function [120], the safety of cell therapy in preventing and treating cardiac senescence remains controversial.

## 12. Concluding Remarks

Cardiac aging is a hot topic in cardiovascular research. Poor cardiac remodeling and dysfunction due to aging are involved in the development of many cardiovascular diseases. In this paper, we systematically reviewed the metabolic changes during cardiac aging from different perspectives, including energy metabolism, gene and hormone metabolism, and molecular signaling pathway metabolism, and focus on the role of mitochondria and autophagy in cardiac aging. We found that these changes are intertwined networks rather than independent of each other, so it is necessary to have a comprehensive understanding of them. The change of energy metabolism plays a prominent role in the metabolism of cardiac aging. Oxidative stress permeates the metabolic process of cardiac aging, leading to gene mutations, mitochondrial dysfunction, and participates in adverse ventricular remodeling. In addition, mitochondria should be given priority in the study of cardiac aging, which is also an important target for correcting or slowing down myocardial aging or improving age-related adverse cardiac remodeling. With the deepening of research, more exploration in the molecular signaling pathway of cardiac aging and delaying cardiac aging by regulating autophagy should be conducted in the future. It will be of great significance to apply the research results in clinical practice to delay myocardial senescence. The analysis of the metabolic changes involved in cardiac aging from different perspectives in this paper helps to understand the process of cardiac aging more comprehensively, and has important significance for how to delay the occurrence of cardiac aging in the future.
